# Near-Surface Mounted Reinforcement of Sawn Timber Beams-FEM Approach

**DOI:** 10.3390/ma14112780

**Published:** 2021-05-24

**Authors:** Izabela Burawska-Kupniewska, Piotr Beer

**Affiliations:** Institute of Wood Sciences and Furniture, Warsaw University of Life Sciences-SGGW, Nowoursynowska 159, 02-776 Warsaw, Poland; piotr_beer@sggw.edu.pl

**Keywords:** strengthening, NSM, CFRP strip, Scots pine

## Abstract

The demand for timber has increased significantly in recent years. Therefore, reliable tools are needed to predict the mechanical properties of sawn timber, especially for structural applications. Very complex models require a lot of input data for analysis, which cannot always be guaranteed, especially in industrial practice. Thus, a simplified model for material description was developed and assessed with experiments (static bending tests carried out in accordance with the guidelines suggested in the European standard EN 408) and an analytical approach (gamma method according to the guidelines given in the European standard EN 1995). The effective stiffness was calculated as a major parameter, which has an influence on the elements’ behavior. The model included a near-surface mounted (NSM) local reinforcement technique, with CFRP strips of Scots pine timber beams being subjected to bending stresses. It is anticipated that the developed model can be a starting point for the repair engineering field, contributing to decision-making regarding conservation technique selection and range. Next, improvements of the model will provide more and more realistic results for numerical analysis in terms of the obtained failure mechanisms for sawn timber elements.

## 1. Introduction

In terms of the deficit of raw wood material, especially of high-quality, and the general increase in prices, it is justified to take up the issue of using inferior quality wood in construction and using it for structural elements. A material of low quality class, loaded with structural defects, can be strengthened, which will significantly increase its original strength parameters. An important issue, however, is the cost of the reinforcement technique used. The reinforced wooden element must be economically competitive with a full-value element of comparable strength properties, available in the required quantity on the market.

The most important determinants of timber strength parameters is the presence of structural defects. The basic and the most common wood defect is knots [1]. Therefore, the presence of knots and resulting fiber deviations is the basic criterion for the quality classification of wood [2,3,4,5,6].

Single knots with a diameter of less than 5mm may be omitted when making the qualitative classification [7]. On the other hand, knots with a particularly unfavorable location, large size, and occurring in clusters may become the reason for the elimination of sawn timber from construction applications. Therefore, in some aspects, it is more reliable to analyze composite materials, e.g., CLT, where the influence of knots on the mechanical parameters can be neglected. In the model developed for 5-layer CLT panels, the perfect connection between each layer (without taking into account the adhesive joint) and the constancy of the mechanical parameters over the lamella thickness was assumed [8]. A similar assumption was made in the analysis of notched connections in a composite system of two materials: timber–concrete (TC system) [9]. Solid sawn timber was not analyzed, but rather wood-based composite with homogeneous material parameters (LVL—laminated veneer lumber).

The negative effect of knots is manifested in the reduction of tensile strength along the fibers, bending, compression along the fibers, and the modulus of elasticity [10]. This impact is caused, among others, by stress discontinuity resulting from the different orientation of the wood fibers, which results from the different orientations of the branches in relation to the trunk. Additionally, the fibers in the trunk show a specific deviation around the knot. After the branches die back and the knot falls out, there is no longer any continuity between the tissues of the branch and the trunk. Therefore, the knot can be considered to be a hole [11]. Another issue is the deviation of the surrounding fibers in the knot itself in the plane tangent to the log, resulting from natural growth processes. The deflection of the fibers in the tangential plane generates shear stresses and stresses perpendicular to the fibers, even during axial loading [12]. In addition, the reduction in strength is also caused by the deflection of the knot fibers in the plane radial to the log [13]. Due to the complexity of the geometrical aspects of the fiber course, it becomes necessary to introduce some simplifications. The studies of wood on a macro scale show that analysis taking into account the simplification of knots to holes and the characteristics of the angle of fiber deviation only in the tangential plane is sufficiently accurate and does not cause significant errors in the estimation of strength parameters [14,15,16]. An analogous error in estimating the bearing capacity is obtained when performing analyses that do not take into account fiber deviation in any plane and assuming the analogy of the knot and the hole [17]. Other modeling methods using a material with a higher density, fully or partially adhering to the surrounding wood, and with a different orientation of the main material axes show a greater error in the estimation of strength properties [18,19]. Nevertheless, models including fiber deformation around knots are being developed. The assumptions for the analysis were taken from the theory of laminar flow around a still obstacle [10,20]. In one model, steeped elliptic obstacles reflected knots, and the fluid flow path represented fiber deflection [21,22,23,24]. Another model involving flow-grain analogy was suggested by Guindos [25].

There are many models developed to estimate the load capacity of a solid timber element, weakened by the presence of structural defects: usually knots and/or fiber deviation. There is a clear shortage of models representing composite materials, for example, timber reinforced with FRP (fiber reinforced polymers), and even more locally, not along the entire length of the reinforced element.

The aim of this research was to develop a simplified model of NSM locally reinforced sawn timber elements. To achieve this aim, it was necessary to validate a model with the results obtained experimentally and analytically, with such comparisons being made at different levels. Validation was possible by verifying the displacement values, comparing the stress and strain distribution determined analytically on the basis of the data obtained from experimental and numerical investigation, and comparisons of the local bending stiffness related to sections weakened by the presence of knots calculated from such strain distribution [26].

## 2. Experimental Details

### 2.1. Material

The test materials were Scots pine (*Pinus sylvestris* L.) sawn timber beams. The nominal cross-section dimensions of the tested timber batch after drying and planing were 50 mm × 100 mm, the length was 2000 mm. A total number of 60 beams were subjected to the study.

The reinforcement material was 1.4 mm thick CFRP strips (CFRP S&P Lamelle CFK 150.2000). The CFRP was selected as a reinforcement material because of its high strength parameters (Table 1), easy availability, and widespread use in the building industry.

### 2.2. Methods

**Figure 1:**
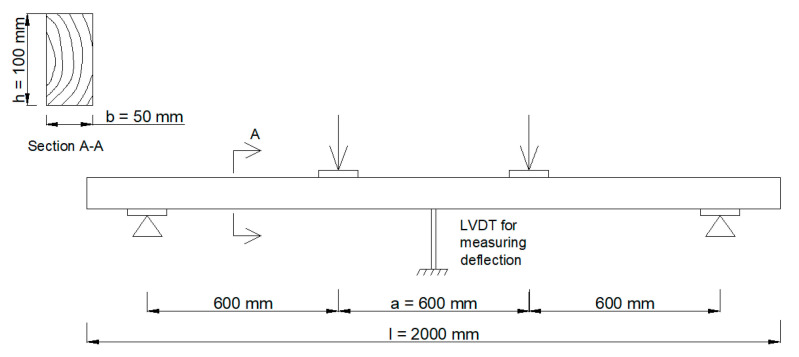
Loading and measuring system for four point bending.

**Figure 2:**
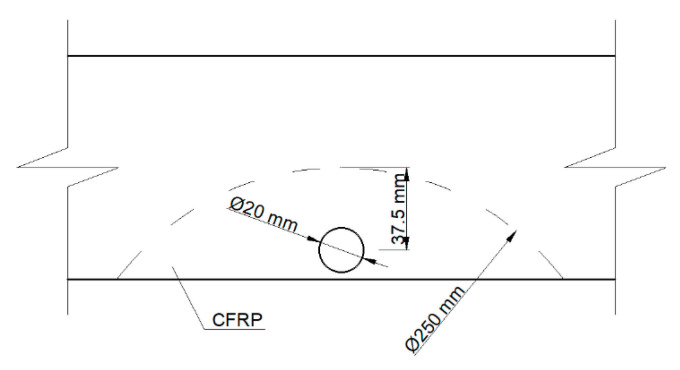
Near-surface mounted (NSM) local reinforcement with a CFRP strip.

All beams, before being reinforced, were tested for elastic range by four point bending (Figure 1) in order to determine the elastic properties of the timber beams and to use the bending stiffness as a characterization of their initial quality [27]. The experimental program assumed testing by bending 60 solid wood samples. Then, all of the samples were weakened with a borehole of 20 mm in diameter, simulating a centric knot in order to investigate the effect of the size of knots in timber beams. The borehole was placed in the middle of the beam’s span, in the tensile zone. Again, samples were tested by four point bending to verify the change in stiffness properties. Then, half of the samples were strengthened by inserting the reinforcement material inside the cross-section and gluing with two-component epoxy resin. The shape of the reinforcement reflected the circular segment. The reinforcement was introduced into a previously prepared slot, made with a circular saw with a diameter of 250 mm (Figure 2). The thickness of the glueline, resulting from the differences in the thickness of the slot and the reinforcement, was 0.2 mm. Due to the fact that the reinforcement method assumes the strengthening of the tensile zone of the cross section, the slot depth was 50 mm. Then, all samples were conditioned for about a month under normal climate conditions, samples were tested again by four point bending until destruction. All bending tests were conducted using a displacement control with a speed rate equal to 3.0 mm/min.

### 2.3. Results

The average density of all samples tested was 563 kg/m^3^ ± 62.5 kg/m^3^, while the MC was 11.5 ± 1.8% (Table 2).

**Figure 3:**
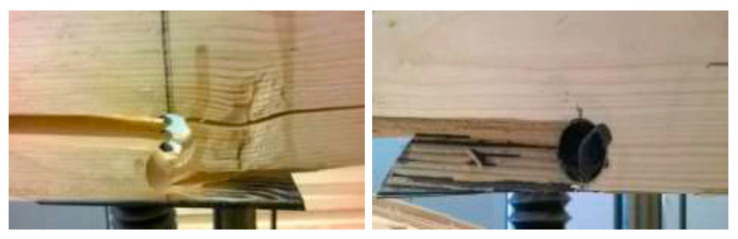
Failure mode in the reinforced samples.

The bending strength of the reinforced samples was significantly higher (by more than 70%, one-way ANOVA analysis) than the bending strength obtained for the samples weakened with a borehole. Typically, the failure was caused by exceeding the shear strength, followed by the crack propagation along the fibers (Figure 3). In view of the destruction occurring in wood within the elasic range, at a high load value (and not in the reinforcing material or in the joint), it can be assumed that the shape, location, and parameters of the reinforcing material were optimal and correctly selected.

Local reinforcement in the shape of a segment of a circle, using a material of adequate strength, is a very effective method for reinforcement. It is related to the increase in the surface of the glue joint and the shape of the reinforcement itself, which results in the lack of local stress concentrations appearing in place of the notches. Additionally, thanks to the possibility of hiding the reinforcement inside the cross-section, and thus the high aesthetics of the method, this type of reinforcement can be used in historical buildings.

## 3. Analytical Analysis

**Figure 4:**
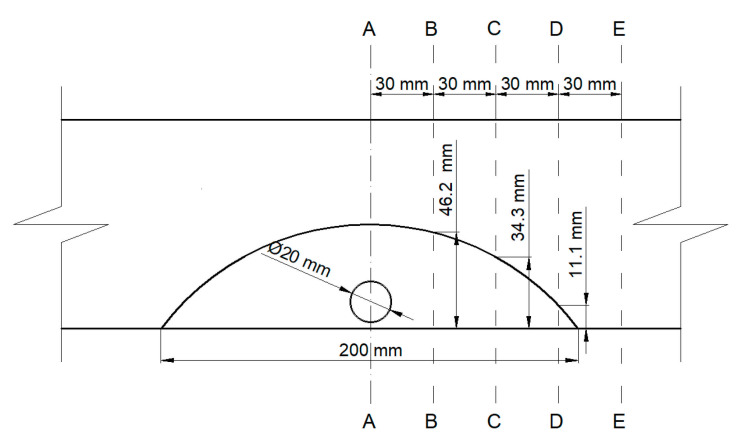
Local reinforcement of a sawn timber element, A-A–E-E–sections were analyzed with the use of the gamma method.

**Figure 5:**
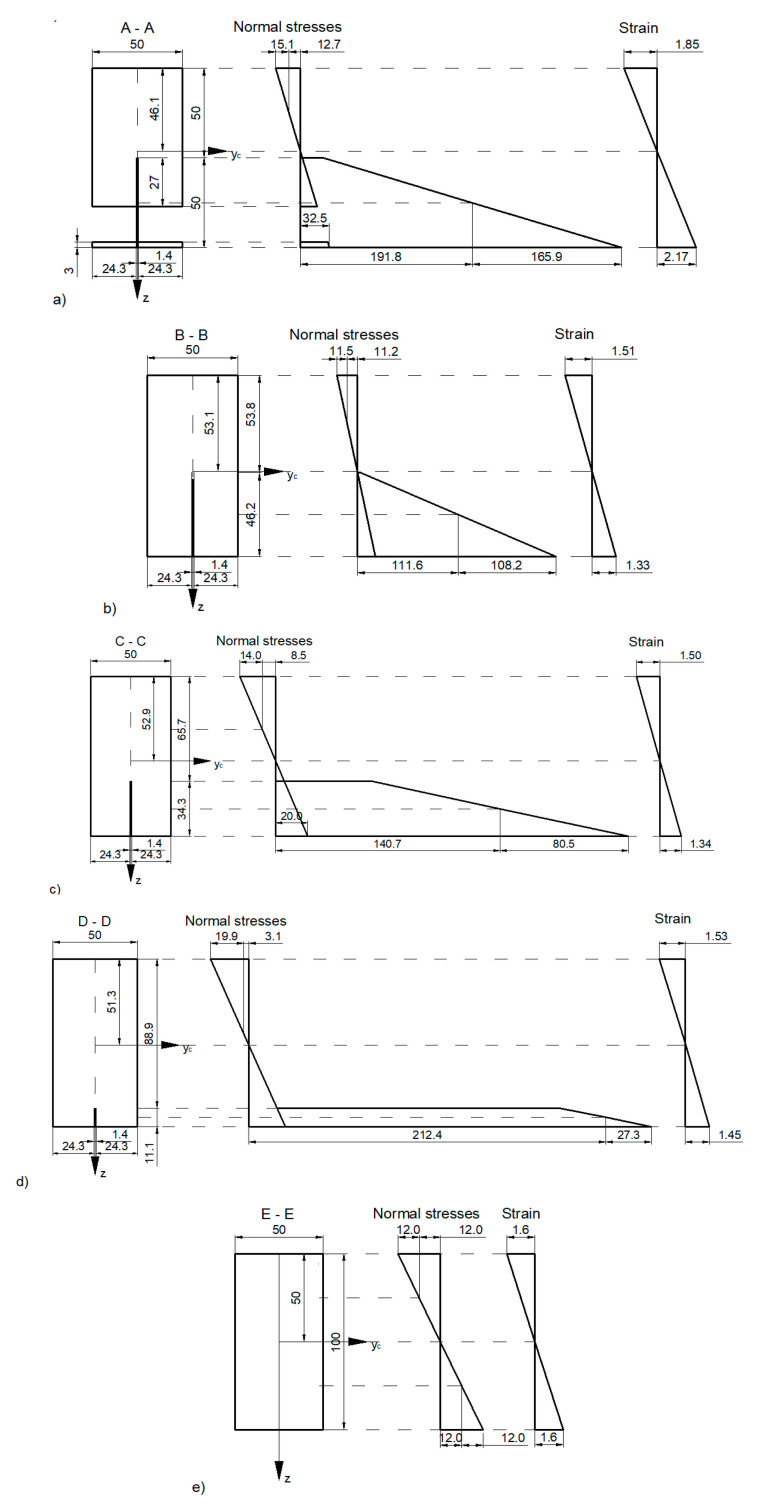
Normal stress and strain distribution in various sections of the NSM reinforced timber beam; whereas: (**a**–**e**) section A-A–section E-E respectively, cross-section dimensions are in (mm), normal stress in (N/mm^2^), and strain in (‰).

The values of stresses and strains were calculated according to the gamma method [28]. The stress and strain distribution was determined in five sections of the bending element (Figure 4 and Figure 5). The effective bending stiffness can be determined using Formula (1):

(1) $${\left(EI\right)}_{ef}=\overset{n_{i}}{\underset{i=1}{\sum }}\left(E_{i}I_{i}+\gamma_{i}E_{i}A_{i}a_{i}^{2}\right)$$

where:

${\left(EI\right)}_{ef}$—effective bending stiffness [Nmm^2^],

$n_{i}\;$—number of different materials within the section [-],

$E_{i}$—modulus of elasticity of the i-cross section [N/mm^2^],

$I_{i}$—moment of inertia of the i-cross section [mm^4^],

$A_{i}$—area of the i-cross-section [mm^2^],

$\gamma_{i}$—reduction factor [-],

$a_{i}^{}$—distance from the middle plane of the whole cross-section to the middle plane of the analyzed section [mm].

Normal stresses were calculated from Equations (2) and (3):

(2) $$\sigma_{i}=\frac{\gamma_{i}\times E_{i}\times a_{i}\times M}{{\left(EI\right)}_{ef}}$$

(3) $$\sigma_{m,i}=\frac{0.5\times E_{i}\times h_{i}\times M}{{\left(EI\right)}_{ef}}$$

where:

$\sigma_{i}$—normal stresses (component 1) [N/mm^2^],

$\sigma_{m,i}$—normal stresses (component 2) [N/mm^2^],

$h_{i}$—height of a given section [mm].

Strain value can be calculated using Equation (4):

(4) $$\varepsilon =\frac{M\times z_{1}}{{\left(EI\right)}_{ef}}$$

where:

ε—strain [‰],

$z_{1}$—distance from the neutral axis of the section to the point for which the deformations are calculated [mm].

## 4. FEM Analysis

Numerical tests were carried out in the Abaqus 6.13 environment (Dassault Systemes, Waltham, MA, USA) using the following assumptions and input data:

(5) $$E_{1}:E_{2}:E_{3}\approx 20:1.6:1$$

(6) $$G_{12}:G_{13}:G_{23}\approx 10:9.4:1$$

(7) $$E_{1}:G_{12}\approx 14:1$$

where:

$E_{1}$—modulus of elasticity in the longitudinal direction (x axis) (N/mm^2^),

$E_{2}$—modulus of elasticity in the radial direction (y axis) (N/mm^2^),

$E_{3}$—modulus of elasticity in the tangential direction (z axis) (N/mm^2^),

$G_{12},\;G_{13},\;G_{23}$—Kirchhoff modules in the xy, xz, and yz directions, respectively (N/mm^2^).

The values of the Poisson coefficients and the yield limits of Scots pine wood were adopted on the basis of literature reports [29,30]. The created FEM model refers to the internal reinforcement (CFRP strip) in the shape of a segment of a circle. The FEM model was developed on the basis of three representative real sawn timber elements, weakened with a borehole simulating a knot and internally reinforced with CFRP. The thickness of the applied adhesive joint was 0.2 mm, while the thickness of the reinforcing tape was 1.4 mm. Necessary parameters, such as the beam’s dimensions, bending stiffness, and bending strength, were adopted from laboratory tests; the other remaining parameters necessary for the analysis were taken from literature or from calculations from the available data (Table 3, Table 4, Table 5 and Table 6).

**Figure 6:**
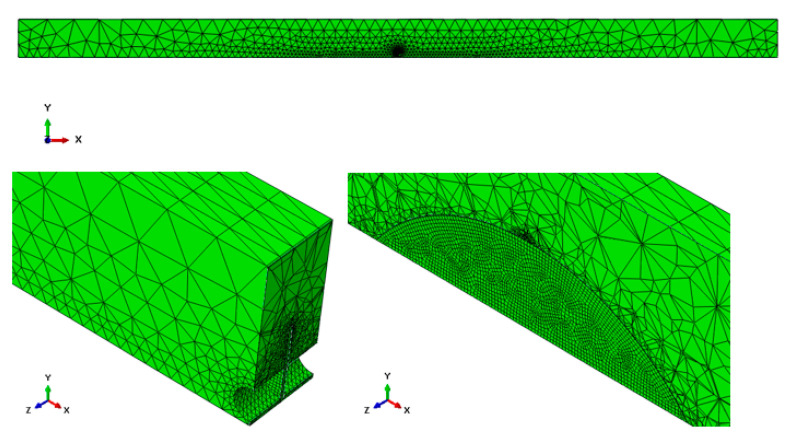
FEM mesh applied to the analyzed element.

Scots pine sawn timber was represented by square tetrahedral, 10-node spatial elements (C3D10). The types of the of FEM elements were automatically selected and optimized by the software. The adhesive joint and the CFRP strip were modeled as four-node coating elements (S4). The FEM model of beam 1 consisted of 58,548 elements and 114,908 nodes (Figure 6), while the models of the remaining beams had a comparable number of elements and nodes to beam 1.

Due to the fact that in bending tests, the crack propagation typically occurs within the elastic range, it was decided not to model the plastic behavior of the pine wood. Based on failure modes analysis (Figure 3), it was assumed that the maximum deformation leading to crack initiation did not exceed the maximum deformation transferred by the CFRP strip.

As a failure criterion, the following principle was applied:

(8) $$\varepsilon_{FRP}\geq \varepsilon_{max}$$

where:

$\varepsilon_{max}$—maximum deformation at which crack initiation occurs [‰].

**Figure 7:**
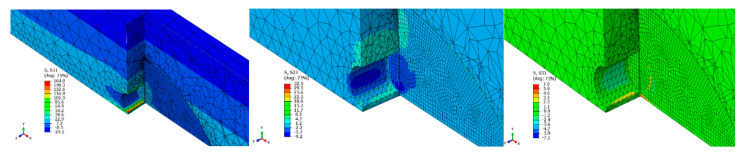
Normal stress distribution in the reinforced element.

**Figure 8:**
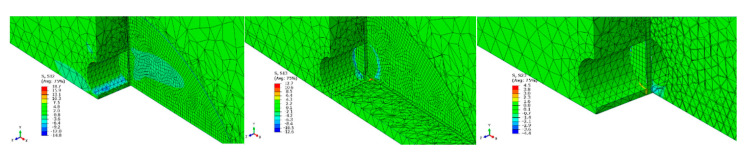
Shear stress distribution in the reinforced element.

Figure 7 and Figure 8 show the individual stress components obtained based on the numerical tests of beam 1. In order to illustrate the results as clearly as possible, the figures show views in various planes.

**Figure 9:**
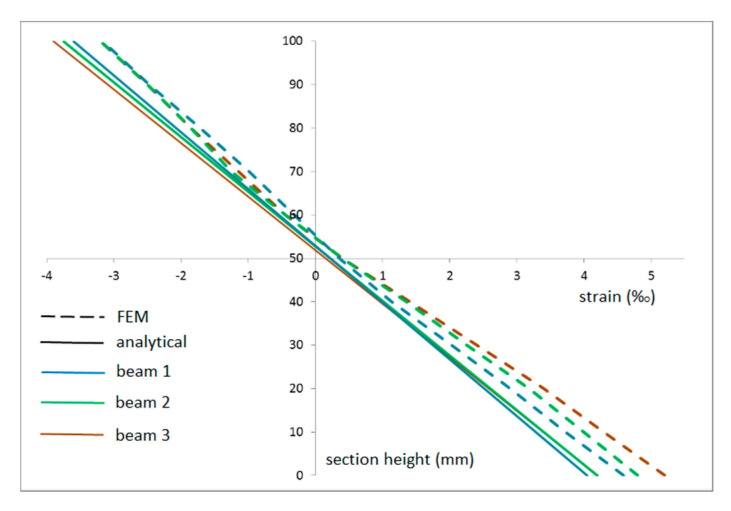
Comparison of the strain obtained on the basis of the FEM and analytical models.

Strain distributions obtained on the basis of FEM analysis and the analytical model (gamma method) showed a very good correlation, although the strain distribution obtained by the gamma method was typically rectilinear (Figure 9). The FEM model was validated in terms of the deflection values at destructive force (Table 7). There were slight differences between deflection values, not exceeding 15% of the actual deflection value obtained during laboratory tests, thus it can be assumed that the FEM model shows a high compliance with the actual behavior of the reinforced element under loading conditions.

## 5. Conclusions

Local reinforcement in the shape of a segment of a circle using a material of adequate strength is an effective method for reinforcement. It is related to the increase in the surface of the glue joint and the shape of the reinforcement itself, which results in a lack of local stress concentrations appearing in the place of the notches. Additionally, due to the possibility of hiding the reinforcement inside the cross-section and thus the high aesthetics of the method, this type of reinforcement can be used in historic buildings.

The validation of the developed model showed a very good agreement in terms of the comparison of the deflection values, stress and strain distribution, and bending stiffness profiles. However, the modeling parameter was set to three sawn timber beams. Therefore, further model validation is needed, including a testing program for subsequent sawn timber elements. Among the reasons for the need for the future development and improvement of the model is the remarkable heterogeneity of the wood structure, which causes a significant variation in the values of the physical and mechanical parameters. When it comes to antique wood, the topic is even more important. The heterogeneity of wood may be additionally increased due to the presence of biological corrosion, cracks, or locally increased wood moisture. Due to the large possible variability of the data, it is difficult to develop a universal model for predicting the properties of wood. It must be noted that under both models (analytical and numerical), the mechanical properties were obtained for each individual beam with laboratory conditions. This approach is unactable in the case of practical and commercial applications. It must also be emphasized that the developed model should not be applied to predict the load capacity when assuming real timber structures, due to the more complex loading conditions, such as cyclic loads. As a consequence of the variety of loading possibilities, more research must be performed in the future to develop more consistent and universal models of internally reinforced timber elements.

Many possibilities become real once a verified model is developed. One application is repair engineering caused by the need for the reinforcement of local cross-section discontinuity. FEM analysis allows us to study timber elements much more easily and thoroughly compared to laboratory experiments. By numerically differentiating the configuration of the critical, weakened area and the crucial range of the local reinforcement, it is possible to successfully study the simultaneous influence of many parameters, which would be unattainable or very expensive when testing in the laboratory.

## Figures and Tables

**Table 1:** Properties of the CFRP strips (data provided by S&P Polska, Malbork, Poland).

Property	CFRP Strip
density (kg/m^3^)	1500
Young’s modulus (N/mm^2^)	>165
tensile strength (N/mm^2^)	>2800

**Table 2:** Laboratory test results (standard deviation is given in parentheses).

Property	All Samples	Weakened Samples	Reinforced Samples
density (kg/m^3^)	563 (62.5)	556 (69.7)	561 (55.3)
bending stiffness (Nm^2^)	51,993 (9557)	48,140 (9688)	46,446 (8641)
bending strength (N/mm^2^)	-	24.4 (7.5)	41.9 (7.7)

**Table 3:** The yield limits of Scots pine wood [30].

Parameter	σ_11_	σ_22_	σ_33_	σ_12_	σ_13_	σ_23_	σ^0^
N/mm^2^	15.5	3.6	3.6	6.0	6.0	3.0	15.5

**Table 4:** Material parameters of the sawn timber beams.

Beam/Parameter	E_1_ N/mm^2^	E_2_ N/mm^2^	E_3_ N/mm^2^	μ_12_ -	μ_13_ -	μ_23_ -	G_12_ N/mm^2^	G_13_ N/mm^2^	G_23_ N/mm^2^
Beam 1 46 × 100 × 1995 mm^3^	13,500	1080	675	0.42	0.37	0.47	964	906	96
Beam 2 47 × 100 × 2002 mm^3^	13,050	1044	653	0.42	0.37	0.47	932	876	93
Beam 3 47 × 100 × 2002 mm^3^	10,663	853	533	0.42	0.37	0.47	762	716	76

**Table 5:** Material parameters of the epoxy resin [data provided by Havel Composites].

Parameter	E N/mm^2^	μ -
	3000	0.3

**Table 6:** Material parameters of the CFRP strips [data provided by S&P Polska].

Parameter	E_1_ N/mm^2^	E_2_ N/mm^2^	E_3_ N/mm^2^	μ_12_ -	μ_13_ -	μ_23_ -	G_12_ N/mm^2^	G_13_ N/mm^2^	G_23_ N/mm^2^
	165,000	10,000	10,000	0.3	0.3	0.03	5000	5000	500

**Table 7:** Comparison of the deflection values obtained on the basis of the laboratory and numerical tests.

Beam/Parameter	Destructive Force (N)	Deflection (mm)	
		Experimental	FEM
Beam 1	11,030	19.1	17.2
Beam 2	11,369	23.6	20.3
Beam 3	10,214	27.2	24.6

## Data Availability

The data presented in this study are available on request from the corresponding author.
